# Lipid metabolism in myeloid cell function and chronic inflammatory diseases

**DOI:** 10.3389/fimmu.2024.1495853

**Published:** 2025-01-22

**Authors:** Ayaka Ito, Takayoshi Suganami

**Affiliations:** ^1^ Department of Molecular Medicine and Metabolism, Research Institute of Environmental Medicine, Nagoya University, Nagoya, Japan; ^2^ Department of Immunometabolism, Nagoya University Graduate School of Medicine, Nagoya, Japan; ^3^ Institute for Advanced Research, Nagoya University, Nagoya, Japan; ^4^ Institute of Nano-Life-Systems, Institutes of Innovation for Future Society, Nagoya University, Nagoya, Japan; ^5^ Center for One Medicine Innovative Translational Research (COMIT), Nagoya University, Nagoya, Japan

**Keywords:** lipid metabolism, immunometabolism, autoimmunity, cancer, metabolic disease

## Abstract

Immune cells adapt their metabolism in response to their differentiation and activation status to meet the energy demands for an appropriate immune response. Recent studies have elucidated that during immune cell metabolic reprogramming, lipid metabolism, including lipid uptake, *de novo* lipid synthesis and fatty acid oxidation, undergoes significant alteration, resulting in dynamic changes in the quantity and quality of intracellular lipids. Given that lipids serve as an energy source and structural components of cellular membranes, they have important implications for physiological function. Myeloid cells, which are essential in bridging innate and adaptive immunity, are sensitive to these changes. Dysregulation of lipid metabolism in myeloid cells can result in immune dysfunction, chronic inflammation and impaired resolution of inflammation. Understanding the mechanism by which lipids regulate immune cell function might provide novel therapeutic insights into chronic inflammatory diseases, including metabolic diseases, autoimmune diseases and cancer. (143 words)

## 1 Introduction

Chronic inflammation has been shown to be the underlying pathogenesis of various diseases. In addition to autoimmune diseases known to be caused by immune abnormalities, immune cell infiltration and aberrant cytokine production are observed in the focal points of metabolic diseases such as obesity and atherosclerosis, as well as in cancer and neurodegenerative diseases. Immune cell differentiation or activation induce metabolic reprogramming in cells, which in turn influences cellular function. This strong link between dysregulation of systemic or intracellular metabolism and immune cell dysfunction, termed immunometabolism, is an emerging field providing critical insights into the pathogenesis of chronic inflammatory diseases (1). In particular, myeloid cells including macrophages, neutrophils and dendritic cells, serve as the first line of defense against pathogens, and play a crucial role in initiating and shaping immune response. They are also involved in tissue repair. Therefore, their dysfunction triggers various pathologies.

Activated immune cells predominantly rely on glycolysis and exhibit downregulated mitochondrial respiration. Although glycolysis is inefficient in ATP production compared to oxidative phosphorylation, it has an advantage in supporting rapid and continuous ATP production because NAD^+^ is regenerated from NADH through the conversion of pyruvate to lactate to sustain further glycolysis. In addition, many of the intermediates of glycolysis serve as precursors for anabolic pathways, including the pentose phosphate pathway which generates NADPH and ribose 5-phosphate for nucleotide synthesis, the hexosamine pathway for glycosylation, and pathways for amino acid and lipid synthesis. Despite the downregulated mitochondrial activity under these conditions, it is not completely lost. Acetyl CoA, which is generated from pyruvate, enters the tricarboxylic acid (TCA) cycle and is converted to citric acid, which is essential for synthesis of fatty acids and triglycerides, as well as cholesterol biosynthesis. These metabolic products are crucial for cell proliferation, cytokine production, pathogen engulfment, and the presentation of antigens to T cells, thereby initiating adaptive immune responses. On the contrary, cells with low energy requirements, such as anti-inflammatory M2 macrophages, regulatory or memory cells, enhances fatty acid oxidation (FAO) and oxidative phosphorylation. This metabolic shift supports the resolution of inflammation and promotes tissue repair. In addition to FAO, glucose utilization remains crucial, as the inhibition of glycolysis prevent M2 macrophage activation (2, 3). This differential metabolic programming in different activation states underscores the crucial role of lipid metabolism in determining cell functions. Lipids are not only an important energy source, but also fundamental components of cell membranes. In this review, we summarize how lipid metabolism is differentially regulated in myeloid cells, contributing to their functions and influencing the development of various diseases.

## 2 Regulation of lipid metabolism

**Figure 1:**
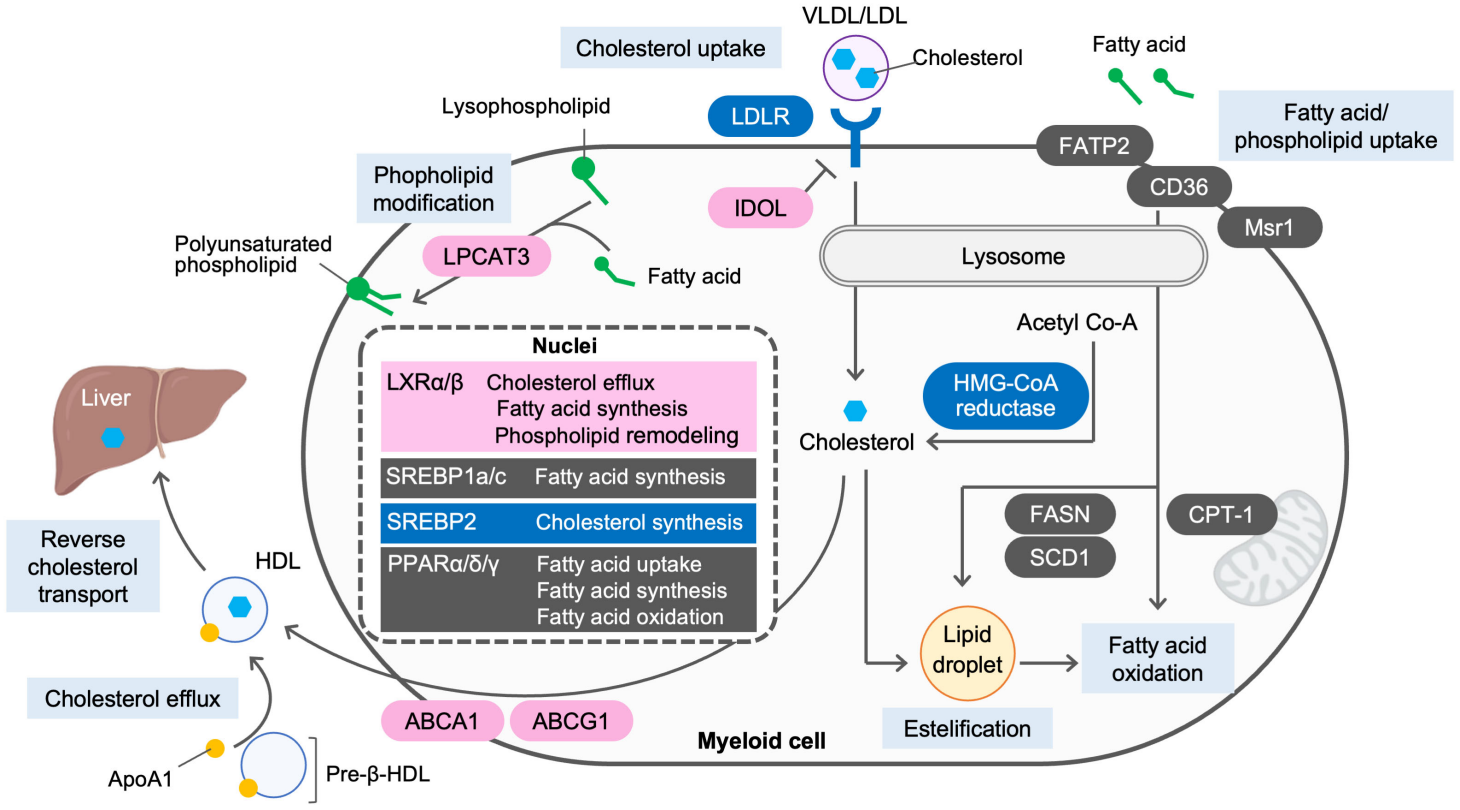
Lipid metabolism in myeloid cells. Lipid homeostasis is regulated by the balance between endogenous biosynthesis, dietary intake, metabolism and elimination from the body. When cholesterol levels are low, sterol regulatory element-binding protein 2 (SREBP2) is activated to synthesize cholesterol from acetyl-CoA and to facilitate cholesterol uptake via low-density lipoprotein (LDL) receptor (LDLR). When cholesterol levels are high, SREBP2 activity is decreased. Instead, activated liver X receptors (LXRs) induce ATP-binding cassette transporter A1 and G1 (ABCA1 and ABCG1, respectively) to accomplish cholesterol efflux to apolipoprotein A1 (ApoA1) or pre-β HDL and reverse cholesterol transport from periphery to the liver. LXRs also induce the expression of inducible degrader of LDLR (IDOL) to degrade LDLR and attenuate cholesterol uptake by the cells. *De novo* lipogenesis is regulated by LXRs, SREBP1a and SREBP1c. Activation of LXRs promotes fatty acid biosynthesis by inducing the expression of SREBP1c. As a key regulator of fatty acid biosynthesis, SREBP1a and SREBP1c induces their targets, including fatty acid synthase (FASN) and stearoyl coenzyme A desaturase 1 (SCD1). Peroxisome proliferator-activated receptors (PPARs) promotes fatty acid uptake via fatty acid transport protein 2 (FATP2) and CD36, modulate fatty acid oxidation or fatty acid synthesis by inducing their targets, such as carnitine palmitoyl transferase 1A (CPT1A), and FASN and SCD1, respectively. Lysophosphatidylcholine acyltransferase 3 (LPCAT3), an enzyme that incorporates arachidonic acid to lysophospholipids and is induced by LXR activation, modulates membrane dynamics by altering phospholipid composition.

Lipid homeostasis is regulated by the balance between endogenous biosynthesis, dietary intake, metabolism and elimination from the body. Several nuclear receptors and transcription factors, including liver X receptors (LXRs), peroxisome proliferator-activated receptors (PPARs), farnesoid X receptor (FXR) and sterol-regulatory element-binding proteins (SREBPs), respond to changes in cellular levels of endogenous lipid ligands and regulate the expression of genes involving in lipid metabolism. Both cellular and systemic cholesterol levels are tightly regulated in a reciprocal fashion by SREBP2 and LXRs. When cholesterol levels are low, SREBP2 is proteolytically cleaved and enters the nucleus to activate transcription of genes controlling cholesterol synthesis, including HMG-CoA reductase, and uptake, such as low-density lipoprotein (LDL) receptor (LDLR), whereas cleavage of SREBP2 is decreased when the cholesterol levels are high (4). Instead, LXRα and LXRβ are activated in response to elevated cholesterol levels and induce the expression of cholesterol transporters ATP-binding cassette transporter A1 and G1 (ABCA1 and ABCG1, respectively) to accomplish cholesterol efflux from peripheral cells such as macrophages. LXRs also activate the transcription of inducible degrader of LDLR (IDOL), and E3 ligase that targets LDLR for ubiquitination and lysosomal degradation, to attenuate LDL uptake by cells (5, 6) (**Figure 1**).

In addition to cholesterol synthesis and efflux, *de novo* lipogenesis is an important component in regulating lipid homeostasis. SREBP1a and SREBP1c, key transcription activators, promote *de novo* lipogenesis by inducing fatty acid synthase (FASN), acetyl-CoA carboxylase (ACC) and stearoyl-coenzyme A desaturase 1 (SCD1) (7). LXRs also stimulate lipogenesis through the direct induction of SREBP1c, FASN and SCD1 (8–11). PPAR family, composed of PPARα, PPARδ and PPARγ, also modulate fatty acid uptake, synthesis and oxidation (12) (**Figure 1**).

## 3 Quantity and quality of lipids in inflammatory responses

It has long been known that macrophages in atherosclerotic plaques produce a variety of inflammatory cytokines and chemokines when they uptake excess lipoproteins and become foam cells (13), suggesting that the quantity of intracellular lipids is associated with inflammatory responses. Indeed, macrophages activated by toll-like receptors (TLRs), induce the accumulation of cholesterol and triglycerides by promoting the uptake of lipoproteins and free-fatty acids, enhancing lipogenesis (14, 15), and inhibiting lipolysis and cholesterol efflux (15–17). Enhanced synthesis of fatty acid and phospholipid is essential for the development of cellular organelles, including mitochondria, lysosome, endoplasmic reticulum and Golgi, for cell proliferation and proper phagocytosis (18). Consistent with this, immune cells are unable to proliferate when cellular cholesterol is insufficient (19, 20). Also, mice lacking SREBP1a, which is a dominant isoform of SREBPs in macrophages, exhibit defects in lipogenesis and thereby decreased phagocytotic ability (21). These mice also show suppressed inflammasome activation and reduced IL-1β production in macrophages, making them resistant to endotoxin shock and systemic inflammatory responses induced by cecal ligation and puncture (22). TLR3 or TLR4 activation strongly induces cholesterol 25-hydroxylase (Ch25h), an enzyme that hydroxylates cholesterol to generate 25-hydroxycholesterol (25-HC) (23). Notably, 25-HC antagonizes SREBP processing, thereby suppressing inflammasome activation in LXR-independent manner, despite being one of the ligands for LXRs (24, 25). Rather, LXR target gene expression, including ABCA1 and ABCG1, and cholesterol efflux are downregulated during inflammatory activation (17, 26). Considering that Ch25h is induced by type I interferons downstream of TLR3 or TLR4, the effect of 25-HC may occur in a late phase of TLR activation, in response to suppressed cholesterol efflux and increased intracellular cholesterol levels. One possible explanation for the antagonism of 25-HC against SREBP is the increased activity of the oxysterol-metabolizing enzyme SULT2B1 and the ABCC transporter, which leads to the elimination of oxysterol ligands for LXRs, thus reducing cholesterol efflux and maintain cellular cholesterol amount (27). LXR-dependent gene expression is also crucial for attenuating inflammation and survival of macrophages and neutrophil (28, 29). In fact, systemic LXR deficiency results in increased susceptibility to inflammatory responses in atherosclerosis, *Listeria monocytogenes* infection and dermatitis (29–32). Intriguingly, viral infection decreases cholesterol biosynthesis while paradoxically increasing cellular cholesterol levels by upregulating cholesterol import and downregulating cholesterol efflux, leading the activation of the stimulator of interferon genes (STING) intracellular DNA sensing pathway (33). A defect in SREBP cleavage-activation protein (SCAP), a key regulator of SREBP, or the silencing of SREBP-2 leads increased production of antiviral cytokines in a STING-dependent manner (33). Given that STING protein resides in the ER, where SCAP/SREBP sense cholesterol, it is likely that STING signaling is activated in response to decreased cholesterol levels in the ER membrane.

While lipids accumulate in inflammatory cells, lipolysis is more prominent in anti-inflammatory cells. Lysosomal lipolysis and subsequent FAO, and mitochondrial oxidative phosphorylation is critical for M2 macrophage activation (14). Additionally, since M2 macrophages play an important role in anti-parasitic immunity, inhibiting lipolysis can impair the elimination of the intestinal helminth parasite *H. polygyrus* (14). Similarly, enhancing FAO and thereby reduction of triglyceride content in macrophages—by overexpressing PPARγ coactivator 1β (PGC1β), a key transcriptional coactivator of oxidative metabolism, or carnitine palmitoyl transferase 1A (CPT1A), the rate-limiting enzyme in mitochondrial FAO—reduced ER stress and the production of inflammatory cytokines (34, 35). On the contrary, suppression of FAO by PGC1β knockdown impairs alternative macrophage activation and rather enhances the production of inflammatory cytokines (35).

Changes in lipid quality, particularly fatty acid composition of phospholipids and cholesterol content, have been shown to determine the biophysical properties, such as fluidity and curvature, of the membrane, thereby influencing various immune cell functions, including cell proliferation, cytokine production, phagocytosis and antigen presentation (36). Wei et al. demonstrated that the deletion of FASN in macrophages resulted in impaired inflammatory responses, which is attributed to decreased membrane cholesterol levels, which in turn disrupts protein trafficking to lipid rafts (37). Correspondingly, activating ABCA1-dependent reduction of membrane cholesterol by LXRs has been shown to modulate membrane lipid organization and preventing the localization of TLR4 adaptor molecules to lipid rafts, thereby impairing downstream MAPK and NFκB signaling (38, 39). Recent comprehensive lipidomic studies have revealed that the activation of macrophages with TLR agonists reprograms cellular lipid composition. In particular, MyD88-dependent TLRs induce *de novo* long-chain fatty acid synthesis in SREBP1c- and SCD1/2-dependent manner, which suppresses prolonged inflammation (40–42). Consistent with this, Hsieh et al. reported that mice lacking SREBP1c exhibit accelerated clearance of *Staphylococcus aureus* (41). The reprograming of lipid composition by various TLRs occurs in distinct, albeit partially overlapping, manners. For instance, TRIF-dependent TLRs attenuate *de novo* long-chain fatty acid synthesis through autocrine type I IFN signaling. Such alterations in lipid quality occur not only at the plasma membrane but also in the endoplasmic reticulum (ER). Even a slight increase in ER cholesterol content reduces the transcriptional activity of SREBP1/2, inhibiting of fatty acid and cholesterol synthesis, while a decrease in ER cholesterol enhances SREBP activity, driving lipid synthesis (43). Lysophosphatidylcholine acyltransferase 3 (LPCAT3), an enzyme that incorporates arachidonic acid to lysophospholipids and is induced by LXR activation, has been reported to contribute to lipid synthesis by altering ER phospholipid composition. This alteration increases SREBP1c activity, thereby promoting fatty acid synthesis. Increased LPCAT3 expression and the abundance of polyunsaturated phospholipids in membranes also ameliorate ER stress in hepatocytes in the setting of obesity (44–46). These findings underscore the crucial role of lipid metabolism in immune cell function.

## 4 Lipid metabolism in inflammatory diseases

### 4.1 Metabolic diseases

As chronic overnutrition progresses to obesity, immune cells, including macrophages, are infiltrated into adipose tissue (47). In obese adipose tissue, macrophages are activated by saturated fatty acids produced by adipocytes, shifting them towards a pro-inflammatory phenotype. This shift is associated with the development of metabolic disorders such as diabetes, dyslipidemia, and fatty liver disease, as well as an increased risk of atherosclerotic diseases (48–50). PPARγ, a sensor of fatty acid, acts as a master regulator of macrophage polarization. Genetic deletion of PPARγ in macrophages results in decreased FAO and alternative activation while increasing the production of inflammatory cytokines, making them more susceptible to obesity and impairing glucose metabolism (51). Furthermore, Ferrante and colleagues demonstrated that in obese adipose tissue, macrophages induce lysosomal biogenesis and consequently accumulate lipids within the cells, leading to an increase in macrophage number rather than macrophage activation (52). Recent single-cell transcriptomics studies demonstrate that adipose tissue macrophages are highly heterogeneous (53, 54). Among the macrophage populations in obese adipose tissue, lipid-associated macrophages (LAM) form a cluster with a distinct transcriptional signature related to lipid metabolism and phagocytosis, and their numbers increase during the progression of obesity. Trem2 null macrophages lack the majority of the LAM gene signature and mice lacking Trem2 exhibit adipocyte hypertrophy and metabolic abnormalities, suggesting that LAMs play a protective role against adipose inflammation and systemic metabolic dysregulation (53). On the other hand, scRNA-seq analysis of CD45^+^ cells from atherosclerotic aorta revealed that macrophages with high Trem2 expression display osteoclastic gene signature, in addition to genes related to lipid metabolism and phagocytosis, suggesting that these macrophages are associated with calcification in atherosclerotic lesion (55). The scavenging receptor CD36 is one of the genes commonly expressed in LAM in obesity, atherosclerosis and cancer (56). Mice lacking CD36 have been reported to show increased insulin sensitivity, decreased inflammation in adipose tissue and resistant to the development of atherosclerosis (57–59). Although these reports do not accurately reflect the role of CD36 in macrophages due to the use of global knockout mice, subsequent study by Moore and colleagues demonstrated that the activation of inflammasomes in macrophages by CD36 is an underlying mechanism (60). Given that cholesterol crystals are endogenous activators of inflammasomes, oxidized LDL taken up via CD36 might form crystals rather than be esterified, thereby activating inflammasomes and exacerbating atherosclerosis (61–63). These findings highlight that managing metabolic diseases through lipid regulation may not only correct systemic lipid metabolism but also improve immune cell functions to mitigate inflammation and metabolic dysregulation.

### 4.2 Cancer

While lipogenic pathway is essential in maintaining the immune cell function, aberrant lipid accumulation can impair tumor immunity and promotes tumor progression. For instance, it has been reported that dendritic cells from tumor-bearing mice or cancer patients have increased lipid accumulation due to enhanced lipid uptake via the scavenger receptor Msr1, which subsequently impairs the ability of presentation of tumor-associated antigens to T cells, partially through triggering ER stress (64, 65). Similarly, myeloid-derived suppressor cells (MDSCs) that are pathologically activated neutrophils and monocytes with immunosuppressive activity and tumor-associated macrophages (TAM) have also been shown to have increased lipid uptake and accumulation via fatty acid transport protein 2 (FATP2) and CD36, respectively. This lipid accumulation supports their proliferation, thereby suppressing tumor immunity and contributing to tumor progression (66–68). Mechanistically, the upregulation of FATP2 increases incorporation of arachidonic acid and leading to the synthesis and secretion of prostaglandin E2 (PGE_2_), and PGE_2_ fuels tumorigenesis by expanding immunosuppressive cells and inhibiting cytotoxic immune cells (67, 69, 70). Moreover, these tumor-associated myeloid cells actively utilize FAO rather than glycolysis to meet their energy demand. Elevated FAO and mitochondrial oxidative phosphorylation result in producing reactive oxygen species and oxidized lipids, which further disrupt tumor-associated myeloid cell function and promote tumor progression (65, 71). Notably, FATP2 inhibition suppresses tumor growth and enhances the efficacy of immune checkpoint inhibitor in tumor-bearing mice (67). Additionally, lipids in tumor microenvironment serve as energy source for tumor cells. Goossens et al. reported that tumor cells promote membrane cholesterol efflux from TAM through ABCA1 and ABCG1, which in turn enhances IL-4 signaling and supports anti-inflammatory/tumor-promoting macrophage polarization (72). In agreement with this observation, myeloid-specific genetic ablation of ABCA1 and/or ABCG1 has been shown to prevent tumor growth (73, 74).

Targeting lipid metabolism has demonstrated promising anti-tumor effects in pre-clinical models, highlighting its potential for clinical applications. For instance, statins, inhibitors of HMG-CoA reductase and cholesterol synthesis, are widely used for cardiovascular diseases and are currently undergoing clinical trials for cancer treatment (75). Additionally, the inhibition of CD36 using thrombospondin analog VT1021 is being evaluated in a phase I trial for solid cancer (NCT03364400), although another thrombospondin analog, ABT-510, failed to achieve positive outcomes in a phase II trial as a monotherapy. Meanwhile, FASN inhibitor TVB-2640 is under clinical studies for various tumors, including breast cancer (NCT03179904), non-small cell lung carcinomas (NCT03808558). Phase I and II trials have already been completed for oral cancer (NCT02223247) and astrocytoma brain cancer (NCT03032484) have already been completed. The anti-tumor effects of targeting lipid metabolism may extend beyond direct impacts on myeloid cells to include the modulation of lipid metabolism in lymphocytes. Indeed, mounting evidence underscores the importance of lipid reprogramming in lymphocyte function, such as those of T cells and natural killer cells, in regulating tumor progression. While this review focuses on lipid metabolism in myeloid cells, the role of lymphocyte lipid metabolism in tumor progression has been extensively reviewed elsewhere (75–77).

### 4.3 Autoimmune diseases

Autoimmune diseases are a diverse group of disorders characterized by loss of immune tolerance with activation of both innate and adaptive immune system and development of antibodies against self-antigens. Impaired clearance of apoptotic or necrotic cells leads increased exposure of self-antigens, resulting in an activation of self-reactive lymphocytes and a break of self-tolerance. This accumulation of apoptotic cell debris also triggers TLRs and cytosolic nucleic acid sensors, which drives the production of inflammatory cytokines (78, 79). Several lines of evidence suggest a link between systemic and cellular dysregulation of lipid metabolism and the development of autoimmunity. Systemically, cholesterol efflux capacity of high-density lipoproteins (HDL) is impaired in patient with systemic lupus erythematosus (SLE) or rheumatoid arthritis (RA), while serum HDL levels are comparable to healthy individuals (80–82). This impaired HDL function could promote lipid accumulation in myeloid cells. The phagocytosis of apoptotic cells activates LXRs, which induces their target gene Mer, a receptor tyrosine kinase that mediates phagocytosis. This positive feedback loop promotes efficient apoptotic cell clearance and suppresses autoimmunity (83). Furthermore, LXR-deficient mice exhibit defective phagocytosis of apoptotic cells, thereby developing autoantibodies (83), suggesting the importance of LXRs in autoimmunity. In fact, polymorphisms in LXR gene are reported to be associated with SLE in Koreans (84). Subsequent mechanistic analyses revealed that cholesterol accumulation in CD11c^+^ antigen-presenting cells and dendritic cells is a driver of systemic autoimmune disease (85–87). LXR deficiency leads to cholesterol accumulation in antigen-presenting cells, enhancing antigen presentation, T cell priming, and production of B cell activating factor (BAFF), a cytokine that supports B cell expansion and autoantibody production and plays a pivotal role in pathogenesis of SLE (86). Increased BAFF production is likely attributed to enhanced TLR signaling due to cholesterol accumulation in lipid rafts (38) Similarly, the loss of LXR targets, ABCA1 and ABCG1, in dendritic cells promotes cellular cholesterol accumulation and cytokine production through inflammasome activation and increased cell surface expression of granulocyte-macrophage colony-stimulating factor (GM-CSF), leading to autoimmunity (87). Mice lacking Ch25h also exert elevated inflammasome activity and exacerbate experimental autoimmune encephalomyelitis (24).

Interestingly, an expression quantitative trait loci analysis in myeloid cells has revealed that fatty acid desaturase genes are associated with increased susceptibility to RA (88), highlighting the significance of fatty acid metabolism in autoimmunity. For instance, the loss of 12/15-lipoxygenase, an enzyme mediates oxidation of polyunsaturated fatty acids, results in increased phagocytosis of apoptotic cells by inflammatory monocytes. This defect leads to the break of self-tolerance and the development of SLE. Mechanistically, 12/15-lipoxygenase, expressed in resident macrophages, oxidizes phosphatidylethanolamine, which sequesters a soluble ‘eat-me’ signal from inflammatory monocytes onto the plasma membrane of resident macrophages. This process inhibits aberrant phagocytosis of apoptotic cells by inflammatory monocytes (89, 90).

Dyslipidemia is frequently observed in autoimmune disease patients and is a major cause of mortality due to cardiovascular diseases. Prolonged glucocorticoid therapy can exacerbate dyslipidemia, making appropriate lipid management an important issue. Statins, widely used for lipid management, have been reported to improve the disease symptoms in RA patients (91). Preclinical studies further demonstrate that statins attenuate autoimmune pathology in models of autoimmune diseases, such as RA, encephalomyelitis, and SLE by reducing antigen presentation, suppressing cytokine production and inhibiting Th1 differentiation (92). Omega-3 polyunsaturated fatty acids, known for its triglyceride-lowering effects, have also shown anti-inflammatory effect on myeloid cells and direct impacts on B cell differentiation (93, 94). Improvements in lipid metabolism are expected to positively influence autoimmune pathology by simultaneously modulating lipid metabolism and immune cell functions.

## 5 Concluding remarks

Over the past two decades, significant progress has been made in understanding the crosstalk between lipid metabolism and inflammatory responses, as well as their collective impact on chronic inflammatory diseases (**Table 1**). Targeting lipid metabolism has emerged as a promising therapeutic strategy to correct immune dysfunction in these conditions. However, translating these findings into clinical applications requires a deeper understanding of lipid metabolism across diverse immune cell subsets, as the regulatory mechanisms vary depending on cell type and disease context. Another critical question that remains unanswered is whether interventions targeting lipid metabolism directly reprogram lipids within immune cells or if the effects are a consequence of alterations in systemic lipid metabolism. Future research, employing lipidomic analyses and imaging technologies, will enable us to elucidate lipid dynamics across organelles, cells, and organs. These insights will open new avenues for developing innovative strategies to address the unmet medical needs in chronic inflammatory diseases.

**Table 1:** Targeting lipid metabolism in inflammation and chronic inflammatory diseases.

Diseases	Molecule	Function in lipid metabolism	Intervention	Phenotypes	Ref.
Inflammation	SREBP1c	Lipogenesis	SREBP1aKO	Reduced phagocytic activity	(21, 22, 41)
			SREBP1cKO	Resistant to endotoxin shock, accelerated clearance of *Staphylococcus*	
	LXRα/β	Cholesterol efflux	LXRα/β double KO	Increased inflammatory cytokines, susceptible to atherosclerosis, Listeria infection and dermatitis	(29–32, 38, 39)
	SREBP2	Cholesterol biosynthesis	shSREBP2	Increased antiviral cytokines	(33)
			SCAPKO	Protected from hervesvirus infection	
	CD36	Fatty acid transport	CD36KO	Less M2 activation	(14)
Metabolic diseases	FASN	Fatty acid synthesis	Myeloid-FASNKO	Decreased inflammation and ameriolated diet-induced diabetes	(37)
	LPCAT3	Phospholipid modification	shLPCAT3/ Liver-LPCAT3KO	Increased ER stress and hepatic inflammation in obesity	(44–46)
	PPARγ	Lipogenesis	PPARγKO	Less M2 activation, increased susceptibility to obesity and impaired glucose metabolism	(51)
	CD36	Fatty acid transport	CD36KO	Attenuated adipose inflammation and insulin resistant, and resistant to atherosclerosis	(57–59)
Cancer	Msr1	Fatty acid transport	Msr1KO	Increased dendritic cell capacity to stimulate T cells	(64)
	ACC	Fatty acid synthesis	ACC inhibitor	Increased anti-tumor effect of cancer vaccine	(64)
	CPT1	Fatty acid oxidation	CPT1 inhibitor	Attenuated tumor growth, and increased anti-tumor effect of chemotherapy or adoptive cellular transfer	(66, 68)
	FATP2	Fatty acid transport	FATP2KO	Attenuated myeloid suppressor function and tumor growth	(67)
			FATP2 inhibitor	Attenuated tumor growth, and increased anti-tumor effect of checkpoint inhibitor	
	ABCA1/G1	Cholesterol transport	Myeloid-ABCA1KO	Decreased myeloid suppressor cells, and attenuated tumor growth	(73, 74)
			Myeloid-ABCG1KO	Elevated inflammatory effect, and attenuated tumor growth	
Autoimmune diseases	LXRα/β	Cholesterol efflux	LXRα/β double KO	Impaired apoptotic cell clearance, increased inflammation, and exhibited systemic autoimmunity	(80, 83)
	ApoA1	Cholesterol efflux	ApoA1/LDLR double KO	Increased autoantibody	(82)
	ABCA1/G1	Cholesterol transport	Myeloid- ABCA1/G1 double KO	Activated inflammasome and exhibited systemic autoimmunity	(84)
	12/15-lipoxygenase	Oxidation of polyunsaturated fatty acid	12/15-lipoxygenase KO	Impaired apoptotic cell clearance by inflammatory macrophages and developed systemic autoimmunity	(87)
